# Overcorrection of the acetabular roof angle or anterior center–edge angle may cause decrease of range of motion after curved periacetabular osteotomy

**DOI:** 10.1093/jhps/hnaa065

**Published:** 2020-12-21

**Authors:** Shinya Hayashi, Shingo Hashimoto, Tomoyuki Matsumoto, Koji Takayama, Tomoyuki Kamenaga, Takahiro Niikura, Ryosuke Kuroda

**Affiliations:** Department of Orthopaedic Surgery, Kobe University Graduate School of Medicine, 7-5-1 Kusunoki-cho, Chuo-ku, Kobe 650-0017, Japan

## Abstract

The aim of this study was to evaluate the relationship between the correction of radiographic parameters and clinical range of motion (ROM) after periacetabular osteotomy (PAO). Sixty-nine patients with hip dysplasia were enrolled and underwent curved PAO. The pre- and post-operative 3D center–edge (CE) angles, total anteversion (acetabular and femoral anteversion), and radiographic acetabular roof angle were measured and compared with the post-operative ROM. The aim of surgery was to rotate the central acetabular fragment laterally without anterior or posterior rotation. Multiple linear regression analysis demonstrated that post-operative internal rotation at 90° flexion was significantly associated with the post-operative Tönnis sourcil angle (rr = 0.31, *P* = 0.02) and that the post-operative ROM of flexion and internal rotation at 90° flexion were significantly associated with the anterior CE (flex; rr = −0.44, *P* = 0.001, internal rotation at 90° flexion; rr = −0.44, *P* < 0.001). However, we found no association between the lateral CE, femoral anteversion, or total anteversion and the post-operative ROM. We demonstrated that the overcorrection of the acetabular roof angle or anterior CE angle may cause a decrease in the range of motion after curved PAO. Therefore, surgeons need to be careful during surgery to prevent the overcorrection of the weight-bearing area and anterior acetabular coverage of the acetabular fragment to avoid femoroacetabular impingement after PAO.

## INTRODUCTION

Hip dysplasia is one of the most common causes of OA in the Japanese young adult population. More than 70% cases of hip OA in this population are caused by developmental dysplasia of the hip (DDH) [1]. The abnormal characteristics in this disorder, including a shallow acetabulum, acetabular mal-orientation, and high anteversion of the femoral neck, cause hip OA due to instability and increased joint contact pressure [2–5]. Therefore, several types of acetabular redirection osteotomies such as dial osteotomy [6], Ganz/Bernese periacetabular osteotomy (PAO) [7], rotational acetabular osteotomy (RAO) [8] and O’Hara/Birmingham Interlocking osteotomy [9] have been developed for DDH cases with mild degenerative changes in the cartilage; these procedures reduce contact pressure on the cartilage and prevent progressive subluxation and degeneration [7]. Curved periacetabular osteotomy (CPO) was developed as an acetabular redirection osteotomy and was considered a modification of the Ganz/Bernese PAO [7]. The exposure of the peri-acetabulum is similar to that performed in the Ganz/Bernese PAO, but the osteotomy line is similar to that used in RAO [8].

Acetabular retroversion is an acetabular morphology that leads to pincer-type femoroacetabular impingement (FAI) [10, 11]. Pincer-type FAI leads to an early pathological contact between the prominent acetabular rim and the femoral neck, and can cause hip pain and OA [10]. These findings indicate that proper acetabular reorientation is a critical issue to avoid anterior or posterior impingement after PAO. Although the most important purpose of acetabular osteotomy is reorientation of the acetabulum into a normal position, PAO can increase the prevalence of secondary intraarticular impingement of the femoral head and extraarticular impingement of the anterior iliac spines during flexion and internal rotation [12]. Analysis of ROMs needs to be better understood to clarify the effects of such impingement.

Several reports have defined the following planning targets for PAO: (i) adequate femoral head coverage based on the acetabular fragment; (ii) determining the horizontal position of the weight-bearing areas in the acetabular fragment and (iii) medialization of the center of the hip in relation to the ilioischial line [13]. Surgeons often try to reproduce pre-operative planning based on the above three targets and prefer to move the acetabular fragment to the horizontal position of the weight-bearing areas by using fluoroscopy during PAO surgery. Precise acetabular correction may ensure good outcomes of PAO; a thorough understanding of the acetabular morphology is important for the accurate surgical correction of a dysplastic hip. For this purpose, surgeons refer to several radiographic parameters including the center–edge (CE) angle of Wiberg [14] and Tönnis sourcil angle [15]. However, the positioning of the patient can affect these radiographic measurements. Computed tomography (CT) imaging and 3-dimensional (3D) reconstruction offer reliable measurements without the undesirable and disturbing superimposition of bony structures [16]. Therefore, we also measured radiographic parameters by using 3D reconstruction CT data to analyze the outcome of PAO. We recently discovered that the post-operative ROM of flexion and internal rotation were significantly associated with the post-operative 3D-anterior CE angles, and concluded that excessive anterior acetabular coverage caused a decrease in the ROM [17]. However, the relationship between radiographic parameters other than the anterior CE angle and post-operative ROM was still unclear. In this study, we aimed to evaluate the relationship between radiographic parameters and the clinical ROM after PAO. Therefore, we measured the acetabular reorientation angles and compared them with the post-operative ROM.

## MATERIALS AND METHODS

### Patients and surgery

This study included 69 patients (76 hips): 9 men and 60 women. The patients underwent curved PAO (CPO) for DDH between January 2015 and April 2018; all surgeries were performed by two senior surgeons. In this study, we focused on the change in the ROM after the movement of the acetabular fragment. Therefore, no femoral osteotomy or osteo-chondroplasty patient was included in this study to avoid ROM bias. CPO was developed as an acetabular redirection osteotomy and was considered a modification of the Ganz/Bernese PAO [7]. The exposure of the peri-acetabulum is similar to that achieved in the Ganz/Bernese PAO, while the osteotomy line is spherical and similar to that performed in RAO [8]. Therefore, the change in the ROM after CPO may be similar to that occurring after RAO [8]. Pre-operatively, all patients were classified as having grade 0 or 1 OA according to the Tönnis classification [15]. The mean age at surgery was 28.1 years (range: 17–49 years), and the mean follow-up duration was 3.3 years (2–4.3 years).

All patients underwent pre-operative 3D planning with a 100 mm radius sphere using a navigation software (OrthoMap 3D Navigation System; Stryker Orthopaedics, Mahwah, NJ, USA). Curved PAO was performed according to the description provided in our previous report [13]. Briefly, a direct anterior approach with a skin incision of approximately 9 cm was used for surgical exposure. A flexion chisel was introduced into the space between the distal joint capsule and psoas tendon. The direction of the chisel toward the infracotyloid groove was confirmed with fluoroscopy, and osteotomy was performed. A pubic osteotomy was performed just medial to the iliopubic eminence. A C-shaped osteotomy line was marked with a power drill from the anterior inferior iliac spine to the distal part of the quadrilateral surface along the spherical position and direction. After performing a spherical osteotomy, the acetabular fragments were rotated laterally and anteriorly to the position and direction determined by fluoroscopy during the pre-operative planning and then fixed temporarily using a Kirschner wire. Two or three poly-L-lactic acid screws or metal cancellous screws were used to finally fix the reoriented acetabular fragment.

An acetabular fragment was usually moved by rotating the central acetabular fragment laterally, without anterior or posterior rotation.

### Clinical evaluation

Hip function was evaluated using the Japanese Orthopaedic Association (JOA) score, which allocates 40 points for pain, 20 points for ROM, 20 points for walking ability and 20 points for activities of daily living, with a maximum total score of 100 points [18]. The JOA score was evaluated pre-operatively and at the 1-year follow-up. The ROM was measured by two senior surgeons using a goniometer. The UCLA activity score [19] was also evaluated at the 1-year follow-up assessment.

### Imaging evaluation

During PAO, surgeons often try to move the acetabular fragment to the horizontal position of the sclerotic sourcil areas by using fluoroscopy. Therefore, the pre-operative and post-operative acetabular roof angles were measured by using plane AP-view radiographs. The Tönnis sourcil angle is measured by drawing a horizontal line parallel to the transverse pelvic axis, at the most medial edge of the sclerotic sourcil, and then making a second line extending out from the medial edge to the most lateral aspect of the sourcil, not the most lateral edge [15]. The lateral CE angle and anterior CE angle were measured by using CT data.

**Fig. 1.**
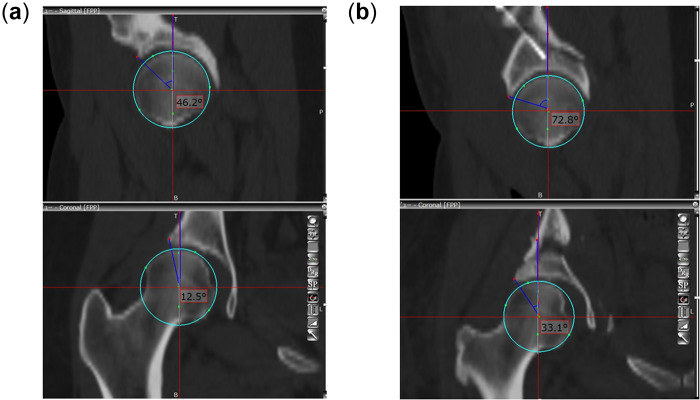
Photograph of the 3D template software (Zed Hip) to measure (**a**) pre-operative lateral (12.5°) and anterior (46.2°) center–edge angles and (**b**) pre-operative lateral (33.1°) and anterior (72.8°) center–edge angles.

All patients were positioned on the CT table in the supine position, and pre-operative CT scans were performed from the pelvis to the knee joint using a 64‐row multislice CT system at our hospital; the obtained image datasets were transferred to a 3D template software (Zed Hip; Lexi, Tokyo, Japan). The software operating window comprised three multiplanar reformation viewers in the coronal, sagittal and axial planes. If the measurement axis is not fixed, CT measurements vary considerably depending on which slice is used in the coronal and sagittal plane. Therefore, we defined the pelvic reference according to the sagittal pelvic tilt in the supine position and both anterior superior iliac spines, which was defined as the functional pelvic plane. The lateral CE angle and anterior CE angle were measured from the coronal and sagittal views through the femoral head center to quantitatively evaluate acetabular coverage in multiple directions (Fig. 1). Acetabular anteversion was measured on the axial view through the center of the femoral head according to the functional pelvic plane on the 3D template. Femoral anteversion was defined according to the method provided by Sugano et al. [20]. Briefly, the femoral neck axis was calculated as the best-fit line connecting slices drawn through a central segment of the neck. Original canal anteversion was defined as the angle between the axis of the neck and a line connecting the epicondylar line.

### Statistical analysis

**Fig. 2.**
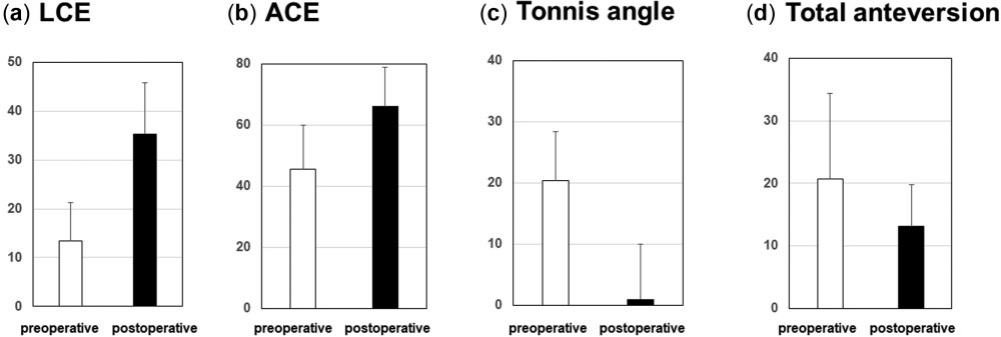
The radiographical outcomes (**a**) pre- and post-operative lateral center–edge angles (**b**) pre- and post-operative anterior center–edge angles (**c**) pre- and post-operative acetabular inclination angles (**d**) pre- and post-operative total anteversion of acetabular and femoral version.

**Fig. 3.**
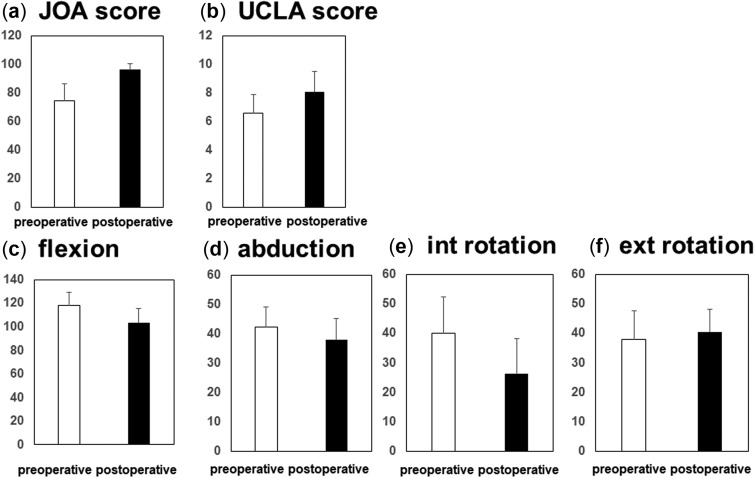
The clinical outcomes pre-operatively and 1 year post-operatively (**a**) JOA score, (**b**) UCLA score, (**c–f**) ROM of (c) flexion, (d) abduction, (e) internal rotation, and (f) external rotation.

All data are expressed as the mean ± standard deviation unless otherwise indicated. Between-group comparisons were evaluated using a Mann–Whitney U test (Figs. 2 and 3). The correlations between groups were analyzed using Pearson’s correlation value (Tables I and II). Multiple linear regression analyses were also performed with the results of the post-operative lateral CE angle, anterior CE, Tönnis sourcil angle, femoral anteversion and total anteversion (acetabular and femoral anteversion) as objective variables and the flexion angle, abduction or internal rotation angle at 90° flexion as explanatory variables. The database was analyzed using the SPSS version 16.0 software (IBM Corp., Armonk, NY, USA). A *P* value <0.05 was considered to be statistically significant.

**Table I.** Relation between Tönnis sourcil angle and acetabular coverage

		Post-LCE	Post-ACE	Post-total anteversion
Post-sourcil angle	Correlation	−0.43	−0.40	0.10
	*P*-value	<0.001	0.001	0.415

**Table II.** Relation among Tönnis sourcil angle, CE angles and post-operative ROMs change

		Post-LCE	Post-ACE	Post-sourcil angle	Femoral anteversion	Total anteversion
Flexion	Correlation	−0.32	−0.50	0.47	0.07	0.25
	*P*-value	0.009	<0.001	<0.001	0.585	0.043
Abduction	Correlation	−0.18	−0.31	0.37	0.01	−0.03
	*P*-value	0.149	0.014	0.003	0.960	0.838
Internal rotation	Correlation	−0.42	−0.65	0.61	0.38	0.42
	*P*-value	<0.001	<0.001	<0.001	0.004	0.001
External rotation	Correlation	0.15	0.01	−0.15	−0.57	−0.13
	*P*-value	0.248	0.976	0.244	<0.001	0.342

### Ethics

The study protocol was approved by the IRBs of the authors’ affiliated institutions, and informed consent for participation in the study was obtained from all participants.

## RESULTS

### Radiographical outcomes

The radiographical outcomes in our study are demonstrated in Fig. 2. The mean values of the pre- and post-operative lateral CE angle changed from 13.4° to 35.4° (*P* < 0.001), those of the anterior CE angle changed from 45.5° to 66.1° (*P* < 0.001) and those of the Tönnis sourcil angle changed from 20.4° to 1.1° (*P* < 0.001). Acetabular anteversion changed from 20.3° to 13.1° (*P* < 0.001). The CE angles, Tönnis sourcil angle and acetabular anteversion showed significant changes (Fig. 2). The mean femoral anteversion was 30.4° (range: 4.4°–59.5°).

### Clinical outcomes

The clinical outcomes are demonstrated in Fig. 3. The mean values of the pre-operative JOA and UCLA activity scores are 72.1 points and 6.4 points, respectively. The mean values of the post-operative JOA and UCLA activity scores are 96.4 points and 8.1 points, respectively. The scores were found to be significantly improved (Fig. 3). The mean values of pre- and post-operative ROM of flexion changed from 118° to 104° (*P* < 0.001), abduction changed from 43° to 38° (*P* < 0.001), internal rotation at 90° flexion was from 40° to 26° (*P* < 0.001) and external rotation at leg extension was from 38° to 41° (*P* = 0.135). The ROM of flexion, abduction at 0° flexion and internal rotation were significantly decreased post-operatively (Fig. 3).

### Post-operative Tönnis sourcil angle is associated with lateral and anterior acetabular coverage angle

We evaluated the correlation between post-operative Tönnis sourcil angle and post-operative acetabular coverage angle. Both lateral CE and anterior CE were significantly associated with post-operative Tönnis sourcil angle (lateral CE; rr = −0.43, *P* < 0.001, anterior CE; rr = −0.4, *P* = 0.001) (Table I). These results indicate that the correction of Tönnis sourcil angle affected the acetabular anterior coverage.

### Post-operative ROM is associated with the post-operative Tönnis sourcil angle

**Fig. 4.**
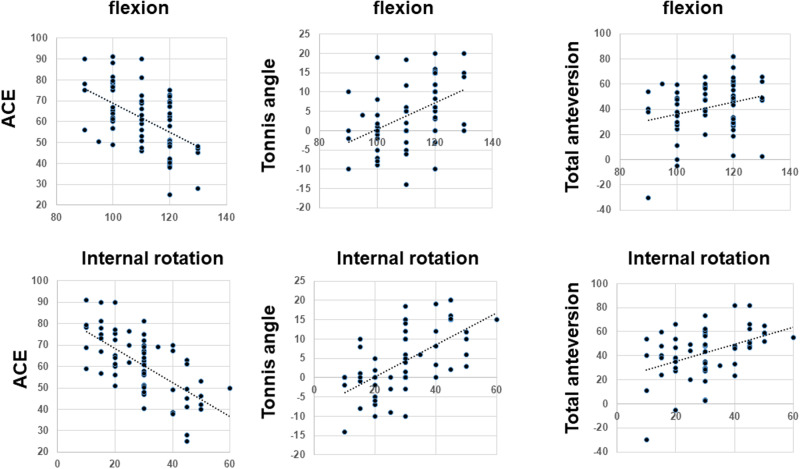
Scatter plots of the relation between radiographic parameters and post-operative ROM.

We evaluated the correlation between the Tönnis sourcil angle and post-operative ROM. The post-operative Tönnis sourcil angle was found to be associated with the ROM of flexion, abduction, and internal rotation at 90° flexion (Table II, Fig. 4). We also demonstrated that the post-operative anterior CE was negatively associated with the ROM of flexion, abduction and internal rotation at 90° flexion (Table II, Fig. 4), and that post-operative total anteversion was associated with the ROM of flexion and internal rotation at 90° flexion (Table II, Fig. 4). Similar associations were found between the anterior CE, Tönnis sourcil angle, or total anteversion and ROM; the Tönnis sourcil angle was found to affect the internal rotation at 90° flexion more than it affected flexion and abduction.

Post-operative lateral CE was negatively associated with the ROM of flexion and internal rotation at 90° flexion (Table II). Femoral anteversion was positively associated with internal rotation at 90° flexion and negatively associated with external rotation (Table II).

### Post-operative internal rotation at 90° flexion was associated with post-operative Tönnis sourcil angle

Post-operative ROM is affected by multiple factors including acetabular coverage and femoral morphology. Therefore, we tested this using multiple linear regression analysis. There was no association between the post-operative Tönnis sourcil angle and post-operative ROM of flexion and abduction. However, we found a significant association between the post-operative Tönnis sourcil angle and post-operative ROM of internal rotation at 90° flexion (rr = 0.31, *P* = 0.02) (Table III). We also demonstrated that post-operative ROMs of flexion and internal rotation were negatively associated with anterior CE (flex; rr = −0.44, *P* = 0.001, internal rotation at 90° flexion; rr = −0.44, *P* < 0.001) (Table III). However, we found no association between lateral CE, femoral anteversion, or total anteversion and post-operative ROM (Table III).

**Table III.** Multiple linear regression analysis

		Objective variables				
Explanatory variables		Post-LCE	Post-ACE	Post-sourcil angle	Femoral anteversion	Post-total anteversion
Flexion	*R*	−0.30	−0.32	0.21	−0.07	0.01
	Standardized *r*	−0.22	−0.44	0.15	−0.07	0.01
	*P*-value	0.122	0.001	0.296	0.541	0.969
Abduction	*R*	0.04	−0.08	0.24	−0.06	0.03
	Standardized *r*	0.06	−0.20	0.34	−0.13	0.09
	*P*-value	0.718	0.202	0.609	0.369	0.711
Internal rotation	*R*	−0.21	−0.37	0.46	0.17	−0.04
	Standardized *r*	−0.14	−0.44	0.31	0.16	−0.54
	*P*-value	0.269	<0.001	0.020	0.123	0.745

## DISCUSSION

Multiple linear regression analysis demonstrated that post-operative ROM of flexion was associated with anterior CE angle and internal rotation at 90° flexion was associated with the anterior CE angle and Tönnis sourcil angle. These results indicate that the anterior CE and Tönnis sourcil angle may be predictive factors for post-operative internal rotation.

Imai et al. demonstrated that an anterior CE angle over 46° may be a probable risk factor for pincer FAI syndrome after an RAO [21]. However, we demonstrated that the anterior CE angle changed from 45.5° to 66.1°. Imai’s study measured anterior CE angles using the false-profile view on radiographs [22]. In the present study, the anterior CE was measured on the sagittal view of CT images through the femoral head center as a parameter of anterior femoral head coverage instead of the vertical–center–anterior angle on false-profile radiographs. The discrepancy between Imai’s study and our study was due to the different sources of measurement. Hamada et al. demonstrated in a simulation study that a lateral CE of 30° and anterior CE of 55° measured by using the sagittal view of CT images produced a coverage similar to that of normal hips, and only the lateral rotation of the acetabulum to achieve a lateral CE of 30° resulted in a larger anterior coverage than that for an anterior CE of 55°, with a decrease of flexion and internal rotation at 90° flexion in a comparison of pre- and post-RAO values [16]. These results were similar to our findings, and we defined an anterior CE of over 55° as anterior over-coverage. Based on our results, the Tönnis sourcil angle was strongly associated with lateral and anterior acetabular coverage. Therefore, overcorrection of the Tönnis sourcil angle may cause anterior acetabular over-coverage, and cause decrease in the ROM of flexion and internal rotation.

Many surgeons often try to achieve a horizontal acetabular weight-bearing area, but the acetabular weight-bearing area is smaller; moreover, it is possible that when the Tönnis sourcil angle is neutral, the lateral CE may still be less than 25° [23]. This may depend on the type of dysplasia. The Tönnis sourcil angle of sloping roof dysplasia may be corrected to neutral, with enough lateral CE angles; however, the sourcil angle of short roof dysplasia, which has a normal Tönnis sourcil angle, may be corrected to neutral, without enough lateral CE [24, 25]. If the fragment of short roof dysplasia is moved into further abduction, the lateral CE may increase; however, the Tönnis sourcil angle would become overcorrected, which increases the risk for femoroacetabular impingement.

We previously reported the univariate analysis of post-operative ROMs and total anteversion of the acetabular and femoral sides and demonstrated that flexion and internal rotation were associated with total anteversion [17]. Our present study also demonstrated similar results, as seen on the univariate analysis (Table II). However, many confounders may affect the ROM. Therefore, we re-analyzed the relation between radiographic parameters and the post-operative ROM by multiple linear regression analysis. Multiple linear regression analysis showed no association between total anteversion and the post-operative ROM.

The limitations of this study, firstly, is that the cohort was likely not large enough to enable a full evaluation of the clinical ROM and acetabular reorientation angle. The acetabular fragment is the only factor which influences the range of motion. Secondly, only acetabular fragment does not influence the ROM. ROM can be influenced by pain, scar tissue and labral irritability. Especially, the external rotation at leg extension may be influenced by capsular scarring because CPO is performed through the anterior approach. Further investigation is required.

## CONCLUSION

Overcorrection of the acetabular roof angle or anterior CE angle may cause a decrease in the ROM after CPO.

Therefore, surgeons need to pay attention to prevent the overcorrection of the weight-bearing area and anterior acetabular coverage of the acetabular fragment during surgery to avoid femoroacetabular impingement after PAO.

## ETHICS APPROVAL AND CONSENT TO PARTICIPATE 

The study protocol was approved by our institutional ethics committee on 8 September 2011 (No. 1219), and informed consent for participation in the study was obtained from all participants.

## CONSENT FOR PUBLICATION

Not applicable.

## AVAILABILITY OF DATA AND MATERIALS

All data generated or analyzed during this study are included in this published article.

## CONFLICT OF INTEREST STATEMENT

The authors declare that they have no competing interests. 

## AUTHOR CONTRIBUTIONS

SHay participated in the study design, drafting of the manuscript and data collection. SHas carried out data collection and drafting of the manuscript. T.M. participated in the data collection and drafting of the manuscript. K.T. participated in the study design and helped to revise the manuscript. T.K. participated in the data collection and drafting of the manuscript. T.N. carried out data collection and drafting of the manuscript. R.K. participated in the study design and helped to revise the manuscript. All authors read and approved the final manuscript.
